# The Use of Pork from Entire Male and Immunocastrated Pigs for Meat Products—An Overview with Recommendations

**DOI:** 10.3390/ani10101754

**Published:** 2020-09-26

**Authors:** Martin Škrlep, Igor Tomašević, Daniel Mörlein, Saša Novaković, Macarena Egea, María Dolores Garrido, María Belén Linares, Irene Peñaranda, Marijke Aluwé, Maria Font-i-Furnols

**Affiliations:** 1 Agricultural Institute of Slovenia, Hacquetova ulica 17, SI-1000 Ljubljana, Slovenia; 2 Faculty of Agriculture, University of Belgrade, Nemanjina 6, 11080 Belgrade, Serbia; tbigor@agrif.bg.ac.rs (I.T.); sasa.novakovic@agrif.bg.ac.rs (S.N.); 3 Department of Animal Sciences, University of Göttingen, Albrecht-Thaer-Weg 3, 37075 Göttingen, Germany; daniel.moerlein@uni-goettingen.de; 4 Department of Food Science and Technology, Veterinary Faculty, University of Murcia, Espinardo, 30071 Murcia, Spain; macarena.egea@um.es (M.E.); mgarrido@um.es (M.D.G.); blinares@um.es (M.B.L.); irene.penaranda@um.es (I.P.); 5 Flanders Research Institute for Agriculture, Fisheries and Food (ILVO), Scheldeweg 68, 9090 Melle, Belgium; marijke.aluwe@ilvo.vlaanderen.be; 6 IRTA-Product Quality, Finca Camps i Armet, 17121 Monells, Spain; maria.font@irta.cat

**Keywords:** entire males, immunocastrates, meat quality, fat quality, pig, meat processing, product quality, boar taint reduction and masking

## Abstract

**Simple Summary:**

Introducing alternatives to surgical castration of pigs bring welfare and economical benefits, but also reveal several quality-related issues. Most important is the presence of boar taint, but also includes low quantity and quality of fat, meat texture and color deviations in addition to inferior water binding properties, most of them negatively influencing meat product characteristics. The present paper highlights the important differences between the conventionally used surgical castrates and the most likely introduced alternatives: entire males and immunocastrates. Based on the review of the available research, the possible reasons for quality alterations are elaborated according to the type of meat product and recommendations for improving product quality or preventing boar taint perception are given.

**Abstract:**

Due to the strong public initiative in Europe and increased regulator focus to mitigate pain, surgical castration of pigs is being gradually abandoned, while the importance of other sex categories like entire males (EM) and immunocastrates (IC) increases. Although beneficial for animal welfare and economics, their use also brings forward several quality problems. Besides the occurrence of boar taint in EM, these include excessive carcass leanness, softer fat, meat color and pH deviations, inferior water holding capacity and increased meat toughness. In this paper, the raw material differences between the male sex categories and their influence on product quality are reviewed, and possible solutions are presented. Using EM for dried or thermally processed products may result in lower processing yields and inferior sensory quality, which may partially be prevented by applying specific processing adaptations. Immunocastration is a viable solution, especially when prolonging the vaccination to slaughter interval. Low to medium levels of boar taint can be effectively managed in most of the meat products, applying procedures like cooking, microbial inoculation or masking (by spices and especially smoking), while highly tainted material can be valorized only by combining various methods and/or with dilution of the tainted meat.

## 1. Introduction

With the future banning of surgical castration without pain relief in Europe, a gradual introduction of alternatives is taking place [1,2]. Production of entire male pigs (EM) or immunocastrates (IC) are both alternatives that enable production systems to phase out surgical castration. Compared to surgical castrates (SC), both alternatives present several important advantages including improved animal welfare (i.e., no painful castration procedure), while (especially in EM), better feed conversion, higher lean deposition, and, consequently higher cost-effectiveness is also evident [3]. At the same time, an EM remains problematic due to more aggressive behavior [4] and particularly due to a potential occurrence of boar taint in muscle and fat. Boar taint, an unpleasant sweaty, fecal- and/or urine-like smell, ascribed mainly to the accumulation of skatole and androstenone in pig adipose tissue [5], is rejected by the majority of consumers. It can be very persistent even in processed meat products; still, several strategies to diminish boar taint exist [6]. Immunocastration (a procedure that requires two vaccinations against gonadotropin releasing hormone) effectively eliminates boar taint shortly (i.e., in several weeks) after effective second vaccination (V2) [7]. Compared to SC, both EM and IC present a metabolically very distinct animal type. Whereas a SC experiences early castration, late castration is practiced in IC, causing rapid metabolic changes (especially in regard to lipid metabolism), resulting in increased feed intake and fast growth [8,9]. On the other hand, EM are characterized by high androgenic potential, low feed intake and high protein deposition abilities [5]. These physiologic differences are reflected in body composition and meat quality.

Apart from the issue of boar taint occurrence, EM may exhibit altered meat quality (as associated with lower adiposity, differences in water holding capacity and higher meat toughness), further influencing meat processing and final product quality [10]. When vaccinated according to common protocols (i.e., 4–6 weeks delay between V2 and slaughter), IC are generally more similar to SC and superior to EM in terms of technological meat quality [10], although published results are far from being consistent [11].

With the increasing market share of EM and IC, the European meat industry will be facing a major challenge of how to adapt processing to the raw material that has altered quality properties. This can only be accomplished by knowing the possible differences between the sex categories as well as possible solutions to overcome them. In view of the mentioned issues, the aim of the present contribution is to review the differences, point out the reasons and (if available) provide recommendations for the use of EM- and IC-derived raw material, either used for fresh consumption or processing to specific meat product types. Being an important issue in EM-derived meat products, possibilities for adding value to meat with boar taint are also reviewed in detail.

## 2. Fat and Meat Quality

### 2.1. Fat Tissue Quality and Composition

It is well known that castration increases fat deposition in pigs [12]. In general, fat saturation in pig carcasses increases with increasing fat thickness, corresponding to higher amounts of de novo synthesized saturated fatty acids (SFA) and monounsaturated fatty acids (MUFA), while diluting polyunsaturated fatty acids (PUFA), which can only be acquired through the diet [13]. In relation to the sex category, the saturation of fat increases in the order of EM < IC < SC (i.e., fat from EM being the least and SC the most saturated) [14], even though differences may not always be significant. Although highly saturated fat may be an issue from a human health perspective, the trait is mostly beneficial for numerous processing and meat product quality aspects [15,16,17] such as oxidative stability, proper development of sensory traits and tissue cohesion.

**Figure 1:**
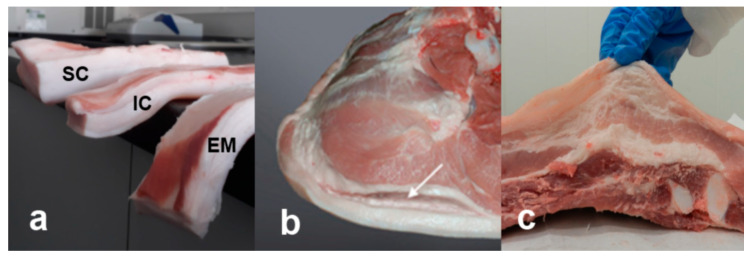
(**a**) Higher fat tissue softness of subcutaneous fat in entire males (EM) compared to immunocastrates (IC) and surgical castrates (SC); inferior cohesion between fat tissue and muscles in the case of EM ham (**b**) and belly (**c**).

In EM, higher PUFA content is observed even at the same fat thickness as in SC, which may be due to the differences in the lipid metabolism during growth (i.e., synthesis [18] and probably also differential fatty acid utilization), governed by the presence of anabolic steroids, that negatively affect lipogenesis and promote lipid expenditure [19], which increase most notably after the puberty onset [5], when the most of the fattening pigs are usually slaughtered. The differences in PUFA content between EM and SC can range from 2% to over 3% points [20,21], with the differences reported in the intramuscular fat (IMF) as much as 7% points (i.e., 26.0 vs. 18.8 g PUFA/100 g lipids in EM and SC, respectively) [22]. Therefore, fat from EM is potentially more prone to oxidation [23], although there is still a lack of literature data or experimental evidence to directly link higher oxidation and fat of EM. Fat tissue of EM contains also more water (3–6% points), more connective tissue and less lipids (5–20% points) than fat of SC, being therefore softer (Figure 1a) and less cohesive (Figure 1b,c) [23,24,25]. This concern (i.e., in terms of attachment between fat tissue layers and to underlying muscles [26]) is especially important when processing products from integral meat pieces such as hams and bellies [17]. Apart from the issue of unsaturated fat, EM also exhibit extensive carcass leanness and very low IMF content [6,23,27] with values below 2% [27] or close to 1% [22], which may be associated with traits like color, juiciness, tenderness, aroma and low processing yields, discussed in the further sections of the manuscript. 

With IC, after the second immunization (usually performed 4–6 weeks prior to slaughter and at the age of 19–20 weeks), which is marked by the decline of testicular steroids, increase in voluntary feed intake, swift change in metabolism from boar-like to more castrate-like and lower basal metabolic rate, the metabolism rapidly changes towards increased fat synthesis [9,28,29]. While not markedly affecting muscle growth (remaining at the similar level), the changes are most notably observed on various carcass fat depots [22]. In this sense, meta-analytical data [30] show IC presented 0.56–2.07 mm thinner backfat than SC and 0.77–1.7 mm thicker than EM. Regarding carcass lean meat content, this is 0.46–1.18 higher in IC than SC and 0.66–1.99 higher in EM than IC. A prolonged period between the effective immunization and slaughter results in diminishing differences in fat characteristics between IC and SC, and larger differences between IC and EM [30]. Backfat thickness, for instance, has been shown to increase linearly proportional to the length of the interval between the second vaccination (V2) and slaughter (SL) at the rate of more than 2 mm in 4 weeks [31]. This effect may be depot-dependent due to the differential response to the stimuli for lipid deposition [32] or the differential onset of maturity of fat depots [33]. As shown by our recent study [34], leaf fat and backfat exhibited higher accretion intensity after immunocastration than other fat depots (such as intramuscular fat (IMF) being a late-developing adipose depot). In parallel to the fat depot quantitative growth, saturation of body fats gradually increases in association with the before-mentioned higher de novo synthesis, and a dilution effect on polyunsaturated fatty acids (PUFA) content [13]. Consequently, a longer V2-SL interval, results in a decrease of PUFA and increase of saturated fatty acids (SFA) and monounsaturated fatty acids (MUFA) content. This transition is relatively fast, whereas some differences between IC and SC fatty acid composition may still exist during the first 3–5 week of V2-SL interval, delaying the slaughter for 6–9 weeks diminishes these differences [35,36,37]. The dynamics of the change may also be dependent on the anatomical location or the fat deposit. For instance, changes in fatty acid composition were shown to occur faster in the belly than in the jowl or in withers backfat [38], or faster in the intramuscular than in subcutaneous fat [34]. At least in the latter case, the reason may be a different tissue lipid content (resulting in faster response in IMF). Intensive IC lipid deposition after V2 can be controlled by dietary modifications. By restricting IC feed allowance to 80% of ad libitum intake, Batorek et al. [39] achieved performance and carcass fatness similar to EM control. Another study on Italian heavy IC [40], however, indicated that feed restriction increases fat unsaturation and iodine value of subcutaneous fat and decreases IMF (making it less appropriate for the production of long-matured dry-cured meat products), but still with no detrimental effects on meat quality (namely oxidation in the muscle). Another possible aspect to consider is the pig breed. As indicated in the meta-analysis by Trefan et al. [41], significant breed effects exist for pork quality traits (e.g., breeds like Duroc and Large White increased and Large White decreased estimated IMF, Meishan breed increased and Large White decreased estimated backfat thickness). The studies with a direct comparison of the male sex category and breed for fat quality traits are, however, scarce and show a limited effect. Park et al. [42], for instance, indicated that the effect of immunocastration on fatty acid composition was relatively consistent in both genotypes (Large White and Duroc crossbreed) evaluated.

### 2.2. Meat Color and pH Value

In the case of meat color and pH, the available literature provides very inconsistent reports [11], Table 1. As to the meat pH, the most valuable are the assumptions of the three available meta-analytical studies [14,29,41], showing either no effect or very low (practically not important) differences between the three male sex categories. Some of the reasons for the inconsistency between the reports are probably the very different carcass treatment conditions (i.e., chilling rate) and conditions prior to slaughter (i.e., mixing, feed deprivation, transport and resting duration) eliciting different levels of (more chronic or acute) stress that may interact with the utilization of muscle glycogen influencing meat pH and consequently other meat quality traits like color and water holding capacity (WHC) [43]. The EM are postulated as being more susceptible to stress due to a higher level of activity and aggressive behavior [4,44]. As demonstrated by the study of Sather et al. [45], if not mixed prior to slaughter, EM exhibited similar muscle pH values (along with other technological meat quality traits) than gilts. On the other hand, preslaughter mixing resulted in higher aggression, more carcass skin lesions, higher occurrence of dark, firm and dry meat (DFD) and higher ultimate pH value. Similarly, higher pH and DFD were observed when EM were held overnight after transport before slaughter in comparison to SC [46]. Newer studies, however, demonstrated no differential response to stress (EM vs. IC or SC [47,48] and IC vs. SC [49]) or even lower stress response of EM in comparison to gilts [50] or SC [39], with little or no notable effect on meat quality [22,48]. It is, however, worth noting here, that the mentioned results refer to different levels and duration of stress (either chronic or acute), which may have very divergent effects on the animal physiology [51].

Similarly divergent (i.e., very study-dependent) are the results for meat color traits (Table 1), which are (being also under the influence of pH) affected by similar slaughter and preslaughter factors [43]. Despite this, there are some indications for either darker (meta-analytical study results [14]), more intensive or more red muscle color in the EM compared to the SC or IC [22]. There may be several reasons for this, including the before-mentioned tendency towards faster glycogen utilization and low intramuscular fat, but also more oxidative muscle metabolic profile and associated higher pigment content (as also indicated by some recent research [52]). Interestingly, IC were often shown (as indicated by meta-analytical data [14,29,41]) to exhibit the lightest muscle (as compared to EM), which is not possible to fully explain. The most likely reasons are somewhat higher IMF or low water holding capacity of the meat [53]. Nevertheless, similarly as for meat pH, the reported color differences are usually small and without much practical importance [14,29,41]. Trials with prolonged (1–21 days) refrigerated storage of IC meat, showed also good color stability (i.e., no sex-related differences in meat discoloration) [54].

**Table 1:** Comparison of pH 45 min after slaughter, ultimate pH and objective color parameter L* between entire males (EM), immunocastrates (IC) and surgical castrates (SC) across different studies.

	pH 45 min		Ultimate pH		Colour L*	
Meta-analytical studies ^1^						
EM–SC	NS	[14,41]	EM > SC	[41]	SC > EM	[14]
			SC > EM	[14]	NS	[41]
EM–IC	NS	[14,41]	EM > IC	[29]	IC > EM	[14,29,41]
IC–SC	NS	[14,41]	SC > IC	[14]	NS	[14,29,41]
			NS	[29]		
Individual studies ^2^						
EM–SC	NS	[22,48,55]	EM > SC	[55]	EM > SC	[52]
			SC > EM	[56]		
			NS	[22,48,52,57]	NS	[22,48,56,57]
EM–IC	EM > IC	[58]	EM > IC	[58,59]	EM > IC	[60]
	NS	[22,55,59,61,62]	IC > EM	[56,60]	IC > EM	[58]
			NS	[22,42,55,57,61,62]	NS	[22,42,56,57,59,61,62]
IC–SC	NS	[22,55,63,64,65,66]	SC > IC	[65]	IC > SC	[66]
			NS	[22,49,55,56,57,63,64,66,67,68]	SC > IC	[64]
					NS	[22,49,56,57,63,65,67,68,69]

Ref. = reference; NS = no statistically (*p* < 0.05) significant differences. ^1^ Meta-analytical studies, including research published until the year 2013. ^2^ Individual studies from the year 2013 until present.

### 2.3. Muscle Water Holding Capacity

As for the ability of the muscle to retain water (which is according to storage or processing conditions measured either as a drip/purge loss, thawing loss or cooking loss, [70], the literature is quite consistent in favor of SC, exhibiting the highest WHC (Table 2). Although more than half of the available studies show no male sex category effect on water holding capacity; still the EM often exhibit inferior WHC in regard to SC. The differences may be quite notable (i.e., cases of up to 45% drip loss differences between EM and SC were reported [52]). With regard to IC, results for WHC are again very study-dependent. Still, several works indicate inferior WHC of IC compared to SC, but with very different positions in regard to EM, although the differences were often without practical importance (Table 2). Reasons for these are not completely clarified. The most probable cause may be elevated muscle protein oxidation (in association with oxidation of unsaturated fats present in EM and IC [24,52] likely causing denaturation, loss of solubility and myofibrillar shrinkage, reducing the ability of muscular structures to bind water [71]. The issue of oxidation may become more evident when heat treatment is applied to the meat. As to our recent study on the meat of EM, IC and SC [20], significant positive correlations between cooking loss and TBARS (thiobarbituric reactive substances), muscle carbonyls (both indicators of either lipid or protein oxidation) or intramuscular PUFA were demonstrated. In the same study, cooking loss was positively correlated with muscle collagen content (with higher collagen amounts found in EM compared to SC muscle and IC taking intermediate position). This correlation was explained by greater water extrusion due to collagen shrinkage during thermal treatment [72]. The consequences of poor WHC are low cooking yields and dry meat (i.e., low juiciness), which are also often reported for EM [73,74,75].

### 2.4. Meat Tenderness

**Figure 2:**
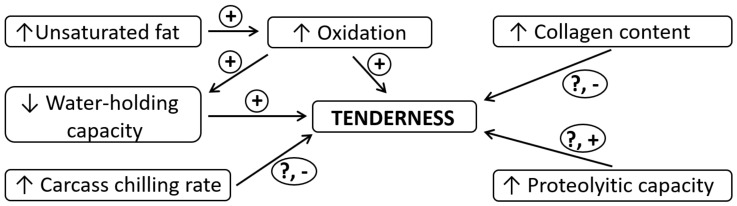
Factors associated or possibly associated to meat tenderness in entire males.

Among the three male sex categories, the highest meat toughness has been reported for EM by meta-analytical [14,29] and several newer individual studies (Table 2). The differences may be quite notable (i.e., EM exhibited 20–25% higher shear force than SC in some studies [22,52]. Similarly, sensory studies indicate EM meat as tougher, drier and less juicy compared to either IC or SC [73,74,75]. As to the reports, the IC are mainly positioned intermediately between EM and SC. Although some studies [14,49,65,68] still demonstrate tougher muscles in IC compared to SC, the differences are mainly low and of lesser practical importance. As to sensory trials, the IC are generally determined to be of very similar eating quality to SC [67,73]. The possible reasons for increased toughness of EM are several (Figure 2), although the issue remains insufficiently characterized [11]. The most probable are the inferior WHC (as associated with water loss due to increased protein oxidation and lower IMF content [22,52]). There were also other possible causes indicated, like higher connective tissue content (as related to higher muscle collagen observed in EM [52,76,77]), differences in carcass cooling rates (as associated to lower fat cover or/and intensity of post-mortem muscle metabolism in EM). However, as to the recently performed research, measuring carcass temperatures after slaughter did not indicate differences between the different sex categories at conventional carcass weights and cooling procedures [50,78]. Conversely, the study of Caldara et al. [64], indicated higher internal carcass temperatures in SC compared to IC due to thicker carcass fat in the former, but the differences were relatively small and without influence on meat quality. There was also no firm relation established between meat tenderness and collagen, the studies of Škrlep et al. [22,52] showed low correlation coefficients between shear force measured on thermally processed meat and collagen content in EM. The reason for that may be increased collagen solubility, reported twice as high in EM compared to SC muscle [52]. Despite higher meat toughness, higher proteolytic capacity of EM muscle has been indicated [11,79,80]. Compared to SC, a higher degree of post mortem degradation of muscle fibers has been indicated for EM, either at the level of myofibrillar fragmentation (higher myofibrillar fragmentation index change in EM, [80]) or at the level of proteomic profile (higher abundance of actin and myosin molecular fragments [52]).

An improvement of meat tenderness in EM is considered possible based on several studies. A study with meat ageing [81] reported that even though EM had tougher meat than gilts at two days post mortem, no significant differences between the sexes were observed after 7 days of ageing. Overall, 7 days of ageing notably improved meat tenderness regardless of the sex category. A subsequent study of Channon et al. [75] investigated the possibility to optimize the acceptability of meat from different of sex categories in relation to cooking methods and selection of pork cuts, concluding that cooking at lower temperatures (i.e., 70 °C vs. 75 °C) and for a shorter time significantly improved meat acceptability (including tenderness). Selection of different pork cuts may present an additional improvement (i.e., shoulder was indicated better than ham or loin muscles). To increase meat tenderness and decreases hardness, rearing strategies like restricted feeding followed by an ad libitum diet were also shown to be effective [82,83,84], promoting compensatory growth response and stimulating protein-turnover and meat proteolytic capacity [85]. The positive effect of compensatory growth was demonstrated for EM by Stolzenbach et al. [86] applying different low protein diets; but although tenderness was improved, the practice had also a negative effect, as EM were not able to fully compensate for the feeding restriction in the early growing period.

### 2.5. Recommendations in Regard to Fat and Meat Quality 

Recommendations on how to manage meat and fat quality issues in EM and SC are resented in the Table 3. 

## 3. Meat Product Quality

### 3.1. Dry-Cured Ham

**Figure 3:**
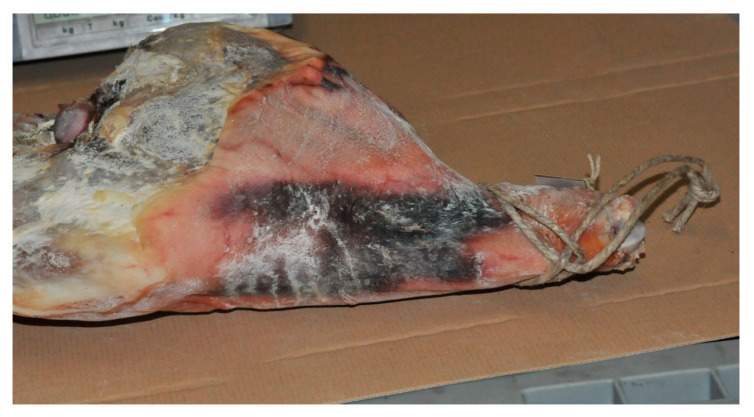
Large subcutaneous hemorrhage visible on the surface of dry-cured ham originating from an entire male pig.

**Figure 4:**
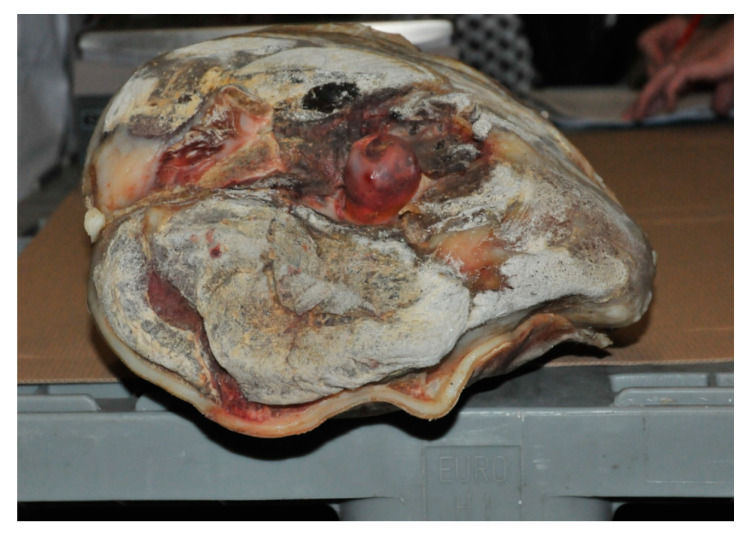
Dry-cured ham produced from entire male pigs; consequences of extensive desiccation and tissue separation on the exterior of the ham are visible.

To produce high quality dry-cured ham, rear legs from older and heavier pigs are preferable, as they are characterized by a higher amount of appropriate fat (i.e., firm and saturated), intensive color and lower proteolytic potential, assuring proper intensity of physical-chemical changes (i.e., water loss, salt uptake, proteolysis and lipolysis) and development of the desired quality characteristics. In addition, for the production of this product type, not only meat and fat quality, but also the outer appearance of the ham is important [17]. Due to more aggressive behavior of EM, higher incidence of skin damage (cuts, bruises, scratches, bites and hemorrhages) may appear on the carcasses [39,92], which are still visible on the dry-cured ham surface (Figure 3) and possibly extend to internal tissues. Whereas superficial defects do not influence ham quality, more profound tissue damage (due to the presence of blood) may increase the danger of bacterial spoilage during processing [17]. Apart from skin and tissue damages and possible boar taint issues, using raw material from EM may result in a series of other issues as it is associated with a low amount of subcutaneous and intramuscular fat or poor water holding capacity [93,94]. These include higher processing loss (due to higher moisture loss), and losses due to tissue separation (Figure 4) resulting in more tissue being trimmed during final product confectioning and deboning), higher intake of salt and a lower extent of proteolysis [95,96,97], a process that is to a certain extent also beneficial for appropriate texture and aroma development [98]. The resulting product exhibits higher sensorial hardness, saltiness and lower juiciness in addition to darker color and less intensive aroma [97,99,100]. In general, EM hams contain less protein and fat and more water than from gilts [101] therefore a decrease in the dry cured ham yield is considered in the following order: SC > gilts> EM. The differences in drying yield (prior to final ham trimming) between EM and SC ranged from 3.3% to 7% points [96,97]. Despite more unsaturated fat of EM hams [95], which is a prerequisite for higher oxidation [71], no differences in oxidative stability (either chemical, i.e., TBARS, or sensorial, i.e., rancidity) has been reported for either EM or IC dry-cured hams even after long-term maturation (up to 15 months) and with no antioxidative additives (like nitrites or ascorbates) applied [97,100]. The reason may be the lower lipid content in either muscle or adipose tissue, which is characteristic for EM. As indicated by Wilson et al. [102], fat content in pig muscles is (opposite to other animal species) positively correlated to lipid peroxidation.

As for producing dry-cured ham from IC, similarity to either EM or SC hams depends on the interval between vaccination and slaughter. In the case of the classical vaccination protocol (4–5 week delay), IC exhibit more similarity to EM than SC hams in terms of processing loss, chemical composition and texture, but with the absence of boar taint [97]. A longer V2-SL interval (i.e., 9 weeks, [100] enlarges the differences towards EM, improving the final product (lower processing loss, salt intake and sensorial hardness). In addition, in heavy male pigs (average live weight 165 kg), vaccination with three doses seems to yield hams with better results than when two doses are used [103]. Immunocastration may also be practiced in female animals, especially when they are intended for dry-cured products. Sensory comparison between hams from IC and SC [104] showed no major differences, apart from a more metallic taste, while dry-cured hams from EM had a clearly different sensory profile. Dry-cured hams from EM appeared to be more adhesive and harder hams than IC and SC hams. Hams from EM had also higher androstenone flavor scores, reducing its sensory quality. 

### 3.2. Dry-Fermented Sausages

Similarly, for dry-cured hams, producing dry-fermented products from EM might bring forward some disadvantages. Due to lower fat content (and possibly also lower WHC) of the raw material, higher processing losses may occur, resulting in drier, harder, more chewy and saltier final products of darker color [105,106,107,108] in addition to the development of some negatively perceived volatile compounds other than boar taint (i.e., aldehydes, furans and sulfur compounds [101]). As to the reports of Corral et al. [105], the use of fat from EM resulted in 3% point higher weight losses compared to the sausages, where fat from gilts was used. Although the raw material originating from EM generally means a higher degree of fat unsaturation (see Section 2.1.), available studies on dry-fermented sausages show no decrease in oxidative stability or excess oxidation (i.e., TBARS levels) either during processing (2 months) or after short time (1–2 week) retail storage [109,110]. The use of previously frozen and thawed raw material was also not increasing the level of EM sausage oxidation compared to SC [110] although this procedure is generally considered to promote oxidative changes [111]. Similarly than in dry-cured hams (see Section 3.1.) the reason may be related to the fact that the use of EM fat results in decreased total lipid content [107] lowering the total oxidation levels. The studies may also differ in their setup (i.e., dietary profile and the type of fat used in the pig diets, differences in the raw material). Corral et al. [107], for instance, reported lower levels of oxidation in the sausages prepared with the use of EM fat than those, from gilts, but also indicated higher saturation of the EM fat used. It is, nevertheless, worth noting, that the formulations of all the mentioned studies on dry-fermented sausages included antioxidants (ascorbic acid, nitrite or nitrate salts) that could have prevented oxidative processes [112] regardless of the fat saturation. As to the available studies published so far, the negative effects of the use of EM raw material may be partly counterbalanced by the use of lower salt addition or microbial inoculation. Both procedures were shown to improve the final product texture and aroma, as they were associated with the increased intensity of proteolytic and lipolytic reactions [106,107], which enhanced the generation of favorable volatile compounds and improved dry-fermented sausage softness [105]. Microbial inoculation also prevented excessive water loss. As demonstrated by Corral et al. [106], surface growth of yeast *Debaryomyces hansenii* regulated water release during the ripening and reduced hardness and chewiness in EM sausages resulting in similar texture to gilt sausages. In another experiment [110], mold-ripening of EM sausages resulted in a 30% softer product, which was close to the level observed in products from SC.

Studies using IC to produce dry-fermented sausages are scarce, to the best of our knowledge, only two studies are available [113,114]. As for the first one [113], dry-fermented sausages from IC were very similar to that from SC raw material either in chemical, microbial or sensory (aroma, texture and taste) traits. Likewise, Stiebing et al. [114] indicated that IC sausages were of comparable quality to those from SC or female-derived raw material, as only small deviations (slightly firmer texture, darker color and higher water loss) were noticed. Similar oxidative stability after long-term (2 months) storage was also indicated (note that antioxidants were added to the formulations).

### 3.3. Cured and/or Thermally Processed Bellies and Bacon 

When using raw material from EM or IC for belly or bacon production, the main issues in this sort of product may arise on the account of lower overall fatness and lower fat saturation [21] as already discussed in the case of dry-cured products. In addition to yielding thin, lean and soft bellies with a possibility of tissue separation, the relatively high incidence of superficial (i.e., skin) damage in EM might be of importance also in this type of product [115,116]. In regard to cured raw bacon, earlier studies [24,117] using pigs slaughtered younger and at lower live weight of around 90 kg, reported 1–8% lower curing yields due to lower brine uptake or lower brine retention, lower fat content and lower water holding capacity of the belly muscles in EM compared to SC. This consequently resulted in a saltier product [115]. No difference in bacon color was reported for EM compared to SC [118]. For consumer acceptance, EM bacon was found to be even better accepted by consumers than that from SC pigs, due to being recognized as more tender and especially more lean [118,119]. It has been suggested that consumers’ main preference is towards leaner pieces [120], even when boar taint is present in the product [119]. In line with raw cured bellies, the studies on thermally processed products [20,121] showed lower processing (i.e., cooking) yield, a lower slicing yield (which may be associated with lower fat saturation and lower tissue cohesion) and lower fat content in EM compared to SC.

For IC, data is available only for thermally processed bacon. Compared to SC, the use of IC resulted in thinner and leaner bellies [122,123]. Although research on raw bellies [124] indicated lower WHC in the case of IC, processing these into bacon resulted in either similar or even higher processing yields. Compared to SC, the product derived from IC was also more acceptable for the consumers. Similar to EM, this was again due to a higher leanness of the pieces [121,123]. Still, a lower commercial slicing yield of IC bacon is reported [21,123] due to the lower belly thickness [125] and lower fat saturation [126,127]. In addition, bacon from IC has been shown to exhibit higher levels of lipid oxidation, but only after long storage times (when frozen for 12 weeks), whereas the oxidation did not exceed sensory thresholds neither it affected normal product shelf life [128]. Results of the trials on prolonging of the V2-SL interval from 5 to 7 weeks concluded that this management procedure mainly eliminates differences in external appearance (i.e., fatness/leanness), slicing loss and slice cohesion in comparison to SC and may be enough to significantly improve bacon quality [123,129].

### 3.4. Other Meat Products

There are only few studies available on the effect of the use of EM or IC in other meat product types (i.e., different kinds of emulsion-type products, cooked or restructured hams or meat). In products from EM, generally no differences compared to SC were reported for either of the processing (i.e., yield), textural or sensorial traits (other than boar taint) quality [130]. For cooked restructured pork roasts from ham muscles, Garcia-Zapeda et al. [131], reported improved texture (higher binding properties and tensile strength) when produced from EM instead of gilts. There were, however, some indications of higher oxidation levels or appearance of oxidation-related negative aromas after thermal preparation or after a relatively long time of storage (i.e., after 6–9 months of freezing) [131,132], although these do not seem to be practically relevant, most probably due to the addition of commonly used antioxidants (nitrites and ascorbates) in the formulations. In contrast, the study of Meier-Dinkel et al. [133] reported texture deviations (the appearance of clearly visible cracks in the filling) of emulsion-type sausages produced from EM, denoting problems during the emulsification and insufficient water binding, possibly caused by decreased water holding capacity and decreased fat saturation. In the IC (again based on a very low number of available studies), raw material quality was reported to be very similar to that of SC. For enhanced pork loins and sausages, the studies of Jones-Hamlow et al. [54,134] did not report any textural, sensory, oxidation (i.e., TBARS) or shelf life (i.e., color and discoloration) differences.

### 3.5. Recommendations in Regard to Meat Products 

Recommendations on how to manage the quality of meat products when produced from EM and IC are resented in the Table 4. 

## 4. Mitigating the Risk of Boar Taint in Meat and Meat Products

**Figure 5:**
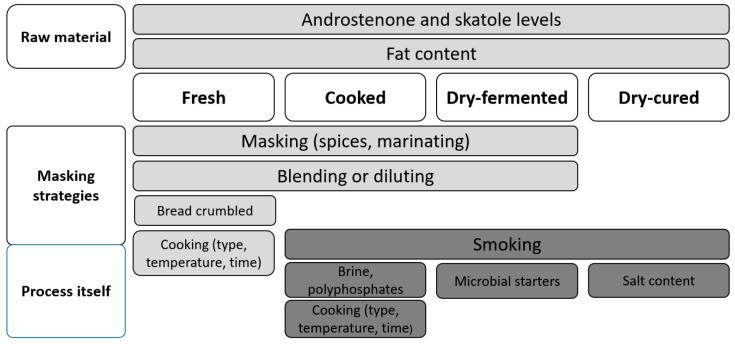
Factors that affect boar taint perception in meat products.

In regard to the mitigation of boar taint in meat products, numerous different strategies exist (Figure 5) being dependent on several factors related to the raw material composition (i.e., androstenone and skatole concentration, fat content), specificities of the processing procedures and masking strategies applied, The factors may be related to the type of the product, governing also the mode of consumption (i.e., hot or cold), which also strongly influences boar taint perception. 

### 4.1. Dry-Cured Ham

It is worth noting, that dry-cured hams are produced in numerous varieties worldwide, either with or without smoking or the addition of various spices [137]. The majority of typical Mediterranean hams are, however, not smoked and produced with a limited amount of added spices, whereas raw material from fattier, more mature pigs with a higher proportion of subcutaneous and intramuscular fat is essential to obtain the desired final product characteristics [17,137]. All afore mentioned factors increase the likelihood of consumers detecting the boar taint aromas and flavors. A positive feature of dry-cured hams is that they are generally consumed cold (lowering the risk of boar taint perception) [6,138,139,140] and that excess subcutaneous fat may be removed before the consumption. However, ham is not consumed in the same way in all countries, for example in Spain, subcutaneous fat is normally consumed with each slice, provoking an increased risk of tainted meat, caused by the accumulation of androstenone and skatole in adipose tissue [99,141].

Although physical–chemical processes during dry-curing may work in the direction of diminishing the boar taint risk, the dry-curing itself was still not found sufficient to prevent the sensation of boar taint in the final product [100,104,141]. Several factors may decrease boar taint including the formation of numerous aromatic compounds as a result of the maturation process that is caused by a certain extent of proteolysis and lipolysis by the proteases and lipases enzymes, respectively [98]. Besides, washing out skatole through a loss of moisture during salting (as being partly soluble in water), evaporation [17,142] and oxidation (triggered by the oxidation of fat and pro-oxidative effect of salt [99,140]) may occur, interacting with other aromatic compounds and masking the boar taint [143]. On the other hand, product desiccation during the processing increases the concentration of boar taint compounds [144]. Boar taint was thus evident in hams even after long processing times (i.e., 15 months) and indicated by either chemical determination of skatole and androstenone or by sensory evaluation by a panel trained in boar taint (i.e., higher incidence of sweat, fecal, persistent or off-flavor sensations [97,99,100,145]). Bañon et al. [99] reported that boar taint in dry-cured hams was sensorially identifiable from 2.0 ppm of androstenone and 0.12 ppm. Font-i-Furnols [146] reported that samples of dry-cured ham with androstenone exceeding 0.5–2.0 μg/g adipose tissue (depending on the study) were unacceptable. Based on Čandek-Potokar et al. [147], the reduction during the processing amounted to 20 and 36% for skatole and androstenone, respectively. In the study by Škrlep et al. [100], a similar reduction during the curing of ham was obtained, but it was not enough to mask boar taint [100]. In both studies, the reduction was higher in the case of androstenone, probably because initial skatole levels were relatively low. 

Although a certain extent of proteolysis may be beneficial in terms of aromatic compounds generation and thus boar taint masking, this may not be the case if the process is too intensive. A positive interaction of boar taint and the meat proteolytic potential (i.e., higher steroid level, being associated with higher protein turnover and thus higher proteolysis [5]) may emerge as a further issue. Kaltnekar et al. [79] reported that increased proteolytic potential was characteristic for EM hams with high levels of boar taint. An increased proteolytic process has been associated with a softer texture and the formation of negative aromatic compounds [148,149], which may add to the perception of off-flavors. The negative effect was especially evident in hams with reduced salt content, since the salt is very important for the stability of cured ham during the initial stages of processing, and low levels of this could further increase proteolysis [150] and the absence of masking potential of foreign flavors by salt [151].

### 4.2. Dry-Fermented Sausages

Similar to dry-cured ham, the dry-fermented products are usually consumed cold, which gives them some advantage in lowering the risk of boar taint perception [140,152].

Still, the process of dry-fermenting alone has not been proven sufficient to mask boar taint in sausages completely [109,153]. This was shown regardless of the microbial culture or additives applied during the processing. The issue is especially problematic when boar taint concentrations are present at higher levels. For instance, in Spanish chorizos (dry-fermented sausage produced with the addition of paprika and garlic) originating from EM with low to medium androstenone content (0.5–0.8 ppm) did sensorially not differ from the same product originating from SC. On the opposite, those containing more than 1.0 ppm androstenone were scored negatively by the sensory panel [108,109]. For most of the dry-fermented products it is characteristic to lose over 30% weight during the process [154]. In general, the loss of water during the drying of sausages may only increase the absolute concentration and thus the perception of boar taint compounds [153]. It was also reported that androstenone (being a relatively stable molecule) [155] did not change during the fermentation process, and to a smaller extent skatole reduction in dry-fermented salami was reported [144]. There may still be some degree of masking by aroma compounds achieved by microbial fermentation (i.e., by adding starter cultures like *Lactobacillus sakei*, *Staphylococcus xylosus* or *Debaryomyces hansenii* [106,107,110]) especially in the case of fast acidification [106,143] or the addition of chemical acidifiers like glucono delta-lactone [153], although other procedures or their combination may prove more effective. Conventional additives for meat products (garlic, spices and polyphosphates) may all help to mask androstenone perception in dry fermented sausages. Recent studies [109,156] reported that the use of rosemary powder, at the concentration of 0.12 g/100 g, together with smoking was the most effective in masking the high concentration of androstenone in fermented sausages. The process of smoking, especially when the procedure was optimized (i.e., use of liquid smoke and intensive smoking [143,157]), was found to be relatively efficient. In their trial, Stolzenbach et al. [143] demonstrated that adding liquid smoke effectively prevented boar taint perception (with androstenone levels up to 1.8 ppm), while conventional smoking (as being applied only on the surface) was less efficient. With higher boar taint concentrations (for example 0.6 ppm skatole and 3.6 ppm androstenone [157]) only long intensive conventional smoking of sausages proved effective. In addition to a purely masking effect, smoke containing formaldehyde has been shown to react with skatole, possibly lowering its concentrations in the products [144,158]. However, if the raw material is highly tainted, only dilution may help to reduce it to the acceptable levels. Fermentation and smoking combined, successfully masked 10% of moderately tainted meat diluted with 90% of untainted meat in the production of smoked fermented sausages [145]. Additionally, it was also confirmed that the use of up to 50% of strongly tainted meat in the standard recipe for smoked fermented sausages did not affect the overall liking of this product [133].

### 4.3. Fresh Meat (Primals, Subprimals, Meat Cuts, Fresh Minced Meat Products like Patties or Sausages)

Similar to processed meat, one of the main criteria that determine boar taint in meat is fat content. Meat pieces with a lean character like tenderloin therefore present a lower risk than blade loin or belly [6,140]. Cooked fresh products are usually consumed warm, which strongly increases the probability of a negative sensory experience [140]. In addition, the procedure of cooking itself is problematic, since high temperatures increase the boar taint release in the space and smelling boar taint during the preparation [6]. On the other hand, thermal treatment (i.e., cooking) has generally been indicated to reduce levels of boar taint compounds and thus boar taint flavor [6,140,159], although no firm consensus on the effectiveness of this procedure has yet been reached. Processes like evaporation (both skatole and androstenone are semivolatile compounds, released in the air to higher degree when heated) [151,158] and processes like interactions with emerging chemical compounds (i.e., Diels-Alder and Maillard reaction between endogenous components, compounds of the masking strategy or cooking/frying medium [160,161,162]) may be the reason for lowering boar taint perception in the cooked products. Whereas androstenone is more lipophilic, skatole is to some extent also water-soluble and can be partly washed out in water used for cooking or by purge loss [163,164].

As to the reduction and masking of boar taint, notable differences exist according to the product or cooking conditions. Cooking at lower temperatures was not found to be efficient in boar taint substance reduction, even if a long time procedure was used [165]. Other research indicated cooking in vacuum (sous vide) as a possible method to reduce boar taint perception [166], although the hermetic packaging may also mean that boar taint compounds do not evaporate during the preparation [162]. Open cooking has been shown to be preferred compared to closed cooking [140]. High temperature cooking seems more efficient, but may also negatively influence meat palatability [162,167]. As to the effect of heating methods on the boar taint perception, frying was recognized as more efficient than roasting, baking in the oven, grilling or cooking in vacuum [162], while roasting was also shown to be more effective compared to cooking in water [168].

The efficacy of the methods may depend on processes like generating masking flavor compounds (i.e., aromatic ingredients of olive oil) or blending fat-soluble boar taint compounds in frying oil [169]. When fried in olive oil, boar taint was imperceptible in samples with medium androstenone concentrations (i.e., 0.5–0.7 ppm) [162]. Additional use of masking ingredients like marinades and spices (i.e., especially when containing liquid smoke, oregano, caraway and savory oleoresin, fennel and curry) were confirmed to improve acceptability when cooked [170,171]. Further methods, like embedding tainted meat in bread crumbs (also preserving juiciness) prior to frying were also determined as efficient when being combined with spices (garlic, parsley or curry) or eaten in combination with bread and cheese [166,172,173].

Although intensive long-time smoking can successfully mask higher levels of boar taint, it is appropriate to be used only in some kinds of fresh pork products like sausages and to a certain degree [157]. Another effective solution is blending. For meat patties (containing 30% fat tissue, spiced with pepper and added breadcrumbs), up to 40% of highly tainted EM carcasses (corresponding to 0.4 ppm androstenone in the product) was reported acceptable [174], but only in combinations with low skatole. When skatole was increased (i.e., 0.037 ppm), this halved the acceptable levels of androstenone in the product to 0.2 ppm and allowed the use of no more than 20% tainted carcasses.

### 4.4. Cooked Meat Products (Cooked Ham, Bacon and Emulsion-Type Sausages)

Similarly as in thermally treated fresh meat, factors like fat content and especially consumption temperature, notably influence boar taint sensation. In this category, the products may, however, be consumed either cold or warm, depending on its variety. Even in cooked smoked products like bacon, warm consumption increases the risk of boar taint. For instance, when serving bacon hot, no levels of androstenone and skatole were low enough to avoid boar taint perception, whereas up to 0.9 ppm androstenone and 0.8 ppm skatole were set as threshold levels in the cold product [175]. In the products that are injected with brine prior to processing (i.e., cooked hams and bacon are pumped up to 120% of the initial weight) a positive effect of a certain level of boar taint dilution can also take place [176]. Application of polyphosphates (commonly used as water binding agents in brine-injected, restructured and emulsion-type meat products) was also considered to mask pork off-flavors including boar taint [177] in addition to flavorings, like hydrolyzed vegetable protein and monosodium glutamate, as indicated for frankfurter sausages by Martínez et al. [178].

An introduction of cooking steps during processing is generally acknowledged to decrease the levels of boar taint compounds or boar taint sensation [6,159], although different studies do not totally agree on whether cooking or different cooking conditions (i.e., higher temperature) has a specific effect. For example, androstenone reduction from 29% [176] up to 60% was reported for cooked in comparison to fresh hams, while notable or even complete elimination of skatole in cooked sausages and cooked ham was shown [158,179]. In cooked restructured ham, more than a 40% reduction of androstenone was reported, while cooking resulted in approximately a 30% reduction of androstenone and skatole content in frankfurter sausages [174]. Others report no boar taint elimination in spite of a heat treatment. Tørngren et al. [139] indicated that in smoked cooked ham processing core temperatures above 70 °C had no effect on boar taint perception. Similarly, no influence of cooking temperatures (65 °C; 72 °C or 80 °C) on boar taint was reported for frankfurters [174]. For frankfurters produced with different masking strategies (smoke, flavorings and spices). Martínez et al. [178] indicated no androstenone reduction, but a relatively high (55–58%) decrease in skatole and ascribed this to the Diels-Alder reaction of skatole with any of the components added as masking agents. The inconsistencies between the studies are most probably a result of differing study designs (sensorial or chemical assessment of boar taint), different products or raw material composition (e.g., fat content and additives), processing conditions and different permeability of the casings used (i.e., cooked-in or open-cook procedure preventing or allowing the release of boar taint compounds [6]).

As indicated before, smoke is the most efficient masking (and possibly also reducing) agent for boar taint, when present in low or medium levels, being less effective in more highly tainted products [157,175,178]. Despite attesting the positive effect of conventional smoking or adding different smoke condensates to cooked meat products, the study of Hemeryck et al. [174] reported no interference with the actual levels of boar taint compounds. For cooked meat products from highly tainted material, combining smoke (or liquid smoke) and spices (like pepper, mustard, paprika, nutmeg, coriander and marjoram, in addition to others mentioned in 3.3.) is far more efficient [178]. In combination with cooking and spices, masking boar taint by smoking was effective when the meat used for production had androstenone concentration from 0.4 to 3.6 ppm [108,157], although it may still not be enough in some high-fat products like frankfurters [133]. Two recent studies on sausages, for instance, did not confirm the potential of conventional [180] or liquid smoke [157] as a fully reliable boar taint masking strategy, although boar taint concentration, in addition to the concentrations of smoke, may also be the reason for discrepancies.

When higher levels of boar taint are concerned, the only technique that is proven to have a positive effect on the reduction of boar taint in cooked sausages is dilution (blending). However, this technique has its limits. It was reported that the usage of 50% of tainted meat for the production of cooked sausages was unacceptable, because masking of boar taint at that amount was not successful [178]. Walstra et al. [181] found that it is safe to use up to 25% of tainted meat in the final product. The same researchers suggested that if the cooked sausage is consumed warm, the maximum amount of tainted meat that can be used is 12%. As a conclusion, when it comes to the use of dilution technique to mask tainted meat in cooked sausages, Mörlein et al. [180] suggested that up to one third of tainted raw material can be used in cooked pork sausages. In their experiment, “the worst-case scenario” was created, using highly tainted carcasses (with 3.8 ppm androstenone and 0.3 ppm skatole in backfat) for the production of high-fat frankfurters (20%), while products were served hot to the consumers.

### 4.5. Recommendations in Regard to the Mitigation of Boar Taint in Meat and Meat Products

Recommendations on how to mitigate boar taint in fresh meat and various types of meat products (dry-cured ham, dry-fermented sausages and cooked meat products) are presented in the Table 5.

## 5. Conclusions

This review shows that shifting to EM or IC production may affect processing and the properties of the final products due to the changes in fat and meat quality. The biggest differences may be observed between SC and EM, with the IC are often positioned in-between and/or may present a quality improvement over EM. Nevertheless, good knowledge of the differences between the sex categories (in addition to thorough monitoring of the raw material quality) is essential to overcome the variations that occur and to optimize processing. Management strategies to avoid a decrease in quality need to be considered at farm, abattoir and processing plants. Another important quality parameter is the boar taint, which can be present mainly in EM pork. Applying immunocastration is an efficient strategy to eliminate boar taint. However, even when EM are used, boar taint reduction and masking strategies such as microbial starter cultures, thermal processing, use of spices with distinctive flavor, smoking and dilution with untainted raw material have been proven to be a viable solution to overcome the issues of boar taint and other quality issues. Appropriate sensory consumer testing should be applied to make sure that meat and meat products from EM and IC are (at least) similarly acceptable.

## Figures and Tables

**Table 2:** Comparison of muscle water holding capacity traits (drip loss and cooking loss) and tenderness (shear force) between entire males (EM), immunocastrates (IC) and surgical castrates (SC) across different studies.

	Drip Loss		Cooking Loss		Shear Force	
Meta-analytical studies ^1^						
EM–SC	NS	[14,41]			EM > SC	[14]
EM–IC	NS	[14,29,41]			EM > IC	[14,29]
IC–SC	NS	[14,29,41]			IC > SC	[14]
					NS	[29]
Individual studies ^2^						
EM–SC	EM > SC	[52,56]	EM > SC	[22,52,56,75]	EM > SC	[22,52,75]
	NS	[48,56,57,67]	NS	[48,57]	NS	[48,56]
EM–IC	EM > IC	[57]	EM > IC	[62]	EM > IC	[22,58]
	NS	[22,42,55,56,59,61,62]	IC > EM	[56,58]	NS	[42,56,59,61,62]
			NS	[22,42,57,59,61]		
IC–SC	IC > SC	[49,56,66,68]	IC > SC	[22,55]	IC > SC	[49,65,68]
	SC > IC	[57,64]	NS	[57,64,66]	NS	[22,56,63,64,67]
	NS	[22,55,63,67]				

Ref. = reference; NS = no statistically (*p* < 0.5) significant differences. ^1^ Meta-analytical studies, including research published until the year 2013. ^2^ Individual studies from the year 2013 until present.

**Table 3:** Summary of possibilities to improve fat and meat quality from entire males (EM) and immunocastrates (IC) compared with surgical castrates.

Content	EM	IC
Fat tissue quality and composition	Increasing lipid saturation by using feed with less PUFA [38] and improving carcass fatness and elevating IMF by rearing animals to higher weights [13,15] or applying high energy/protein ratio diets [87]. To be dealt with precaution (risk of boar taint [88] and growth retardation [86]).	Prolonging the V2-SL interval to more than 7 weeks to improve fat quantity and saturation. The delay may be optimized, in order to achieve the most favorable relation between carcass fatness and composition of fat [35,36,37] and, at the same time, assure clearance of boar taint. [89]. Dietary modifications (restriction–realimentation) are possible [39,40].
Meat colour, pH and WHC	No clear differences. General recommendations could be used: optimization of the fasting time prior to slaughter, short transport times, no mixing between unfamiliar animals, optimization of slaughter and chilling procedures [90,91].	No clear differences. General recommendations could be used.
Meat tendernes	Higher meat toughness may be related to decrease in WHC. The same recommendations that for meat colour, pH and WHC may be used. Applying specific rearing methods like restriction–realimentation and different protein/energy ratios, but these may be problematic in regard to EM performance [86]. Other strategies include prolonged storage (ageing up to one week), selection of favorable cuts (shoulder better than leg or loin parts) and optimizing cooking procedures (preparation at lower temperatures and shorter time) [75,81].	Tenderness differences of low practical importance.

PUFA = polyunsaturated fatty acids; WHC = water holding capacity; V2-SL = vaccination to slaughter.

**Table 4:** Summary of possibilities to improve meat products quality from entire males (EM) and immunocastrates (IC).

Content	EM	IC
Dry-cured ham	Close monitoring of raw material (skin damage, tissue consistency and fat thickness) and processing losses; adjustment of salting/ripening time and processing conditions (temperature, air humidity, and circulation) to decrease processing loss and optimize salt content.	Prolonging V2-SL interval to increase similarity to SC and improve processing yield and sensory quality.
Dry fermented sausages	Controlling temperature during mincing of the meat batter [16] to prevent surface coating. Adaptation of processing conditions (time, air humidity, circulation and temperature), salt reduction [106,107] and applying microbial cultures (to decrease water losses and improve aromatic profile) [106,110].	Only slight differences from SC, scarce data.
Cured and/or thermally processed bellies and bacon	Controlling raw material quality and processing yields during individual processing phases. Modification of curing, thermal processing and slicing conditions (including amount of brine injected, duration and temperature) to adapt to thinner and leaner raw material [135,136].	Adjustment of the delay between the second vaccination and slaughter (to over 7 weeks) may help in the modification of leanness/fatness of the product and to avoid negative consequences on processing and slicing yields [123,129].
Other meat products	Controlling raw material quality and composition. Use of antioxidants and water binding agents.	Raw material with similar characteristics to SC.

**Table 5:** Possible strategies to reduce boar taint perception according to the meat product type and boar taint level.

Products.	Boar Taint Level	Masking Capacity without Strategy ^1^	Strategies
Dry-cured ham	Low-Medium	+	Preselection of carcasses. Extra caution on salt reduction and long ripening process needed.
	High	+	Not recommended.
Dry-fermented sausages	Low-Medium	+++	Addition of microbial cultures (i.e., yeast) in combination to a long ripening process [106] or fast acidification [143]. Use of smoke [143,158]. Combining different methods is more efficient.
	High	+	Dilution with not tainted meat [133].
Fresh meat	Low-Medium	+	Use of leaner meat cuts [6,140]. Preparation at home and high serving temperatures not recommended [6]. Cooking for a longer time, at high temperatures and conditions that enable volatilization of boar taint: frying in oil [162]. It is recommended use of spices (especially those with distinctive aromas), their respective essential oils or liquid smoke [170,171] and their combinations [166,172,173]. Masking may be used for products with low to medium boar taint but is never complete.
	High	+	Not recommended. Intensive smoking and dilution [157,174].
Cooked meat products	Low-Medium	++	Open cooking process (allowing evaporation and drainage) should be applied. Combination of smoking and spices [133,178].
	High	+	Dilution (mixing of 1/3 of tainted with untainted meat/fat [180].

^1^ Denotes for the ability of the commonly used processing practice to produce the specific product type; + = low; ++ = medium, +++ = high.
